# Photoperiod-dependent developmental reprogramming of the transcriptional response to seawater entry in Atlantic salmon (*Salmo salar*)

**DOI:** 10.1093/g3journal/jkab072

**Published:** 2021-03-12

**Authors:** Marianne Iversen, Teshome Mulugeta, Alexander C West, Even H Jørgensen, Samuel A M Martin, Simen Rød Sandve, David Hazlerigg

**Affiliations:** 1 Department of Arctic and Marine Biology, UiT -The Arctic University of Norway, Tromsø NO-9037, Norway; 2 Department of Animal and Aquaculture Sciences, Norwegian University of Life Sciences, Ås NO-1432, Norway; 3 School of Biological Sciences, University of Aberdeen, Aberdeen AB24 2TZ, UK; 4 Centre for Integrative Genetics, Department of Animal and Aquaculture Sciences, Norwegian University of Life Sciences, Ås NO-1432, Norway

**Keywords:** smoltification, smolting, osmoregulation, gills, Atlantic salmon, photoperiod, NFAT5, glucocorticoid

## Abstract

The developmental transition of juvenile salmon from a freshwater resident morph (parr) to a seawater (SW) migratory morph (smolt), known as smoltification, entails a reorganization of gill function to cope with the altered water environment. Recently, we used RNAseq to characterize the breadth of transcriptional change which takes place in the gill in the FW phase of smoltification. This highlighted the importance of extended exposure to short, winter-like photoperiods (SP) followed by a subsequent increase in photoperiod for completion of transcriptional reprogramming in FW and efficient growth following transfer to SW. Here, we extend this analysis to examine the consequences of this photoperiodic history-dependent reprogramming for subsequent gill responses upon exposure to SW. We use RNAseq to analyze gill samples taken from fish raised on the photoperiod regimes we used previously and then challenged by SW exposure for 24 hours. While fish held on constant light (LL) throughout were able to hypo-osmoregulate during a 24 hours SW challenge, the associated gill transcriptional response was highly distinctive from that in fish which had experienced a 7-week period of exposure to SP followed by a return to LL (SPLL) and had consequently acquired the characteristics of fully developed smolts. Fish transferred from LL to SP, and then held on SP for the remainder of the study was unable to hypo-osmoregulate, and the associated gill transcriptional response to SW exposure featured many transcripts apparently regulated by the glucocorticoid stress axis and by the osmo-sensing transcription factor NFAT5. The importance of these pathways for the gill transcriptional response to SW exposure appears to diminish as a consequence of photoperiod mediated induction of the smolt phenotype, presumably reflecting preparatory developmental changes taking place during this process.

## Introduction

The gill is the primary site of osmo-sensing and osmoregulatory control in fish (Evans *et al.* 2005; Evans 2010). In both freshwater (FW) and seawater (SW), osmoregulatory systems work to counter the passive diffusion of ions and water across the gill epithelium, and balance plasma osmolality. Euryhaline fish species are defined by their ability to tolerate salinity changes through modulation of osmoregulatory function. In most cases this depends on responses to altered salinity (acclimation), while in a few species groups including salmonids and eels (genus Anguilla), sustained migrations between sea and freshwater are facilitated by preparative changes in osmoregulatory function, forming part of a key developmental life-history transition (Folmar and Dickhoff 1980; Wilson *et al.* 2004; Kalujnaia *et al.* 2007; Stefansson *et al.* 2008).

In Atlantic salmon (*Salmo salar*), this preparatory process is commonly known as “smoltification” or, hereafter, “smolting.” Smolting is photoperiodically controlled so that migration to sea occurs in a spring “smolt window,” when conditions favor juvenile growth (Gross *et al.* 1988). Smolting does not occur before the fish exceed a certain size threshold and is presumed to relate to the capacity of juvenile fish to meet the necessary metabolic demands (Higgins 1985; Kristinsson *et al.* 1985; Metcalfe *et al.* 1988; Skilbrei 1991). During smolting, the juvenile salmon develop traits that will enable them to survive in and exploit the marine environment. Following exposure to short day lengths in winter, the increase of photoperiod in spring induces a hormonal cascade influencing behavior, metabolism, growth, pigmentation, and gill physiology (Duston and Saunders 1990; McCormick 1994; McCormick *et al.* 1998, 2007). In particular, gill physiology changes in order to accommodate the expected shift in environmental salinity and osmotic drive (Pisam *et al.* 1988; Evans *et al.* 2005; Kiilerich *et al.* 2007; Nilsen *et al.* 2007; Tipsmark *et al.* 2009). The mitochondria rich cell (MRC), situated on the gill lamella, is a significant component of osmoregulation (Wilson and Laurent 2002). The MRC is rich in ion transporters, and changes in both morphology and composition in response to salinity (Pisam *et al.* 1988; Hwang and Lee 2007; Madsen *et al.* 2009; Hwang *et al.* 2011; Hiroi and McCormick 2012). Completion of the smolting process requires entry to sea, where SW exposure triggers the final shifts in physiology and behavior (Pisam *et al.* 1988; Lubin *et al.* 1989; Nilsen *et al.* 2007; McCormick *et al.* 2013). Hence, smolting can be considered a two-step process: a FW preparative phase followed by a SW activational phase.

Recently, we performed an RNAseq experiment designed to identify the hallmarks of photoperiodically induced smolting in the gills of the Atlantic salmon (Iversen *et al.* 2020). By comparing RNA profiles from fish raised continuously on constant light with those that experienced a 7-week period of short photoperiod, simulating winter photoperiod, before return to constant light we were able to identify a cohort of novel genes the expression of which is winter-photoperiod dependent. In a second experiment we saw that the length of winter-photoperiod exposure was critical to these genes and growth performance after SW transfer. 

This finding provides a genome-wide analysis of the well-described preparative phase of smoltification, but does not address the issue of further activational changes triggered in smolts during the first few days in SW (Prunet and Boeuf 1985; Handeland *et al.* 1996, 2000; Stefansson *et al.* 2008), hereafter the “SW activational phase.” SW responses are also triggered in juveniles entering SW prematurely, which have not initiated or finished the preparative phase of smolt development (Saunders *et al.* 1985; Stagg *et al.* 1989). Triggers may include osmotic stress due to the hyper-osmotic SW environment as well as direct responses to changes in the concentrations of specific ions either in the gill or in internal organs such as the kidney and intestine (Evans and Somero 2008; Evans 2010; Kültz 2012). However, the specific response is expected to differ drastically between SW-ready smolts and unprepared juveniles (Stagg *et al.* 1989; Houde *et al.* 2018). The importance of SW-exposure for completion of the smolting process and establishment of a SW phenotype is clearly demonstrated by the process of “de-smoltification,” which occurs if migration to SW is prevented and involves a loss of tolerance to SW (Stefansson *et al.* 1998; Arnesen *et al.* 2003).

Gill tissue may respond to SW in at least three possible ways: (i) as a direct response to increased cellular tonicity and altered intracellular ion concentrations (ii) as a direct response via cell surface receptors for SW constituents (*e.g.*, Ca^2+^ perceived via the calcium-sensing receptor, CaSR) (Nearing *et al.* 2002; Loretz 2008; Kültz 2012) and (iii) as an indirect response via hormonal signals (*e.g.*, cortisol, or angiotensin II) which change in response to SW-exposure (McCormick 2001; Kültz 2012). In this context, the “nuclear factor of activated T-cells” (NFAT) family of transcription factors have been the focus of recent interest because of their implication in osmo-sensing and Ca^2+^-dependent transcriptional control (Hogan *et al.* 2003; Putney 2012; Cheung and Ko 2013; Lorgen *et al.* 2017). The NFAT family comprises four subgroups, where groups 1-4 (NFATs c1, c2, c3, c4) are Ca^2+^-stimulated, and the fifth, NFAT5, is regulated in response to extracellular tonicity (Rao *et al.* 1997; Macian 2005; Cheung and Ko 2013). All members share a Rel-like homology domain, and bind to similar binding sites in the regulatory regions of their target genes (Macian 2005).

NFAT5 (also known as osmotic response element-binding protein, OREBP, or tonicity-responsive enhancer-binding protein, TonEBP), is considered the primordial NFAT, as it is the only one found outside the vertebrate group (Hogan *et al.* 2003). NFAT5 regulates the transcription of tonicity-responsive genes such as ion transporters and osmo-protective proteins (Woo *et al.* 2002; Zhou *et al.* 2006; Cheung and Ko 2013). Hypertonic stress increases nuclear import and retention of NFAT5 through changes in phosphorylation state, while hypotonic stress leads to nuclear export (Ferraris *et al.* 2002; Macian 2005; Irarrazabal *et al.* 2010; Cheung and Ko 2013).

Two recent studies in salmon focus attention on the role of NFAT signaling during smolting. Lorgen *et al.* (2015) showed that the salmonid thyroid hormone deiodinase *dio2a* was SW-inducible in gill tissue, and its promoter region was enriched for osmotic response elements (OREs/NFAT5 response elements). A subsequent survey of NFAT5 expression in Atlantic salmon (Lorgen *et al.* 2017) revealed four NFAT5 paralogues, NFAT5 a1 and a2, and NFAT5 b1 and b2. Of these, NFAT5b1/2 gill expression was highly induced by SW exposure. Together these studies suggest that NFAT5b1/2 could coordinate SW stimulated changes in transcription.

In this study, we have extended our transcriptomic analysis of pre-adaptive, photoperiod-induced changes in gill phenotype (Iversen *et al.* 2020) to consider the shifting transcriptional response to SW-exposure. Specifically, we aimed first to assess the extent of the transcriptional response to an acute SW-challenge, and to determine how this is affected by prior photoperiodic exposure. Secondarily, we sought to infer the importance of stress (glucocorticoid) and NFAT-signaling in the shifting pattern of SW-responsiveness. Our data indicate that as salmon develop into smolts these pathways are less activated upon SW-entry.

## Materials and methods

### Fish rearing and experimental set-up

**Figure 1:**
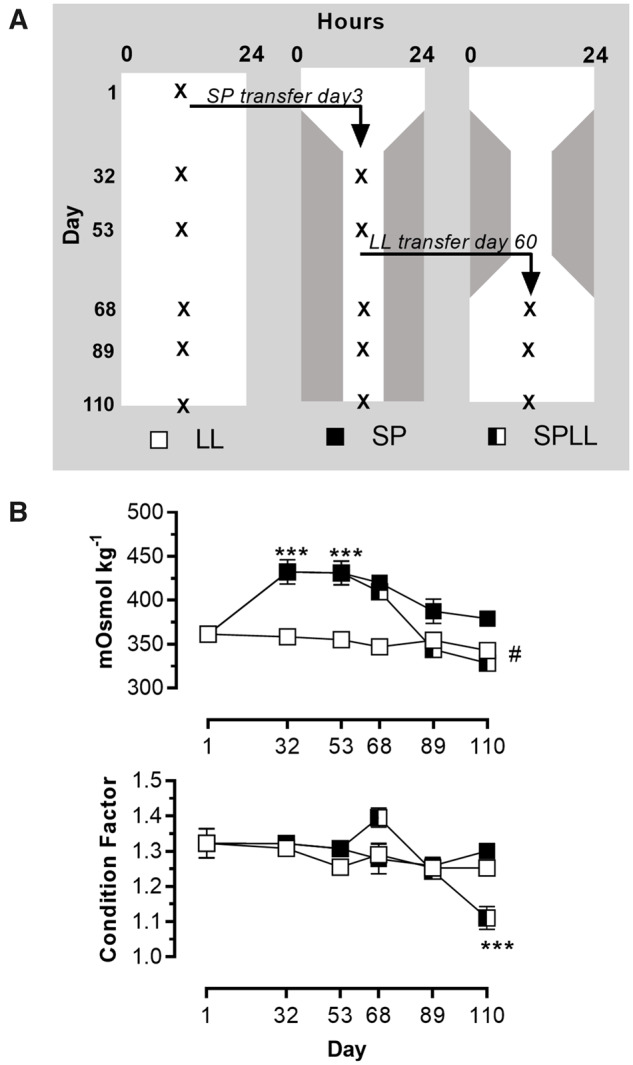
Experimental design and the effect of SW challenge on hypo-osmoregulatory capacity. (A) Experimental set-up showing the three photoperiod treatments. The protocol established groups of fish with three distinctive photoperiodic histories: those raised on constant light throughout life (LL group), those transferred from LL to 8 hours light/24 hours (SP) at day 3 of the study, and maintained on SP to the end of the study (SP group), and those which were returned from SP to LL after 7 full weeks of SP (SPLL group). The white/dark gray shading depicts the periods of light and dark in each 24 hours period over the course of the study. Arrows indicated the points at which tank transfers were made to establish the SP group (day 3 of the study) and subsequently the SPLL group (day 60 of the study). Sampling days are indicated by Xs. (B) Physiological indices of smoltification: upper panel shows plasma osmolality following 24 hours SW challenge, and lower panel shows body condition factor (100 × weight (g)/length(mm)^3). Note re-establishment of acute SW tolerance over last 3 sampling points in SPLL fish, in parallel with a marked decline in CF only in the SPLL group. All data are mean ± S.E.M of *n* = 6 observations; *** = significantly lower values in SPLL fish than in fish from the same group on day 68, *P* < 0.001.

All studies were performed in accordance with Norwegian and European legislation on animal research. The experimental design has been described in detail previously (Iversen *et al.* 2020), and is presented schematically in Figure 1A. 

Briefly, juvenile Atlantic salmon (*n* = 225) of the Aquagene commercial strain were raised at the Aquaculture research station in Tromsø (69.867 N, 18.935E), a bespoke salmonid research facility drawing a natural freshwater supply from the Kårvikelva river (mean/Sd water parameters: pH 6.92/0.12; aluminum 25.5/14.5 µg/ml; calcium 1.88/0.26 mg/ml; potassium 0.29/0.01 mg/ml; sodium 3.60/0.03 mg/ml). Fish were kept on continuous light from hatching until approximately 7 months old, when they weighed on average 49.5 g (s.d. = 7.0 g). Then the fish were divided into two groups of 75 and 150 fish by dip netting and placed in separate 100 l circular tanks in separate rooms (FW, 8.5 °C). The group of 75 fish were kept on LL for the rest of the experiment, while for the group of 150 fish, photoperiod was incrementally reduced over a week until it reached 8 hours light/24 hours (SP). After 7 weeks, half of the fish transferred to SP were moved to a new 100 l circular tank and returned to LL, again by incrementally increasing photoperiod over 1 week (hereafter referred to as the SPLL group), while the remainder continued on SP. The experiment continued for a further 6 weeks. During the experiment the fish were fed continuously and in excess over the eight hours corresponding to day in the SP treatment group, using standard commercial salmon pellets (Skretting, Nutra Olympic 2,0 mm).

### Sampling procedure

Fish were sampled from all tanks on days 1, 32, 53, 68, 89, and 110 (*n* = 6 for each treatment) of the study. At each sampling point another subsample of fish from each of the treatments were transferred to new tanks and put through a 24 hours salt-water challenge [SWC, 100 l tanks, 34‰, salinity, 7 °C, *n* = 6 for each treatment, as detailed in Iversen *et al.* (2020)], starting on the day prior to sampling. All fish were fasted for 24 hours prior to each sampling point.

All fish were terminally anesthetized (Benzocaine 150 ppm) before sampling, after which body mass (±0.5 g) and fork length (±0.1 cm) were measured. In the fish that had been exposed to the 24 hours SWCs, blood was drained from the caudal vein into 2 ml lithium heparinized vacutainers (BD vacutainers^®^, Puls Norge, Moss, Norway), and held on ice. Blood samples were centrifuged at 6,000 × *g* for 10 minutes, and the plasma fraction collected. The plasma was kept frozen at −80°C until analyses for osmolyte content using a Fiske One-Ten Osmometer (Fiske Associates, MA, USA, ± 4 mOsm kg^−1^).

From sampling day 68 onwards, the right operculum was removed, and a gill arch dissected out, on the sampled fish in both groups (directly from FW and after SWC). The primary gill filaments were then cut from the arch and placed in RNAlater^®^ (Sigma-Aldrich, St. Louis, MI, USA). Samples were stored at 4 °C for 24 hours, and then kept frozen at −80°C until RNA extraction.

### RNA extraction and sequencing

RNA extraction, library preparation, and Illumina HiSeq analysis were performed as described previously (Iversen *et al.* 2020).

### Transcriptome analysis of SW-sensitive gene expression

Transcriptome analysis was performed using the Edge R package (version3.14.0) and R (version 3.4.2), run in RStudio (version 1.0.153). Raw counts were filtered (expression threshold CPM > 1 in five or more libraries), and scaled using trimmed means of *M*-values (TMM). Principal component analysis (PCA) was performed on all above threshold genes using The R Stats Package (Stats, ver. 3.4.2) (Love *et al.* 2014). For simplicity and interpretability of the plot, TMM normalized counts for each gene in each sample group (*n* = 6, except for on day 68 SPLL FW where *n* = 5) were averaged before generating the PCA plot.

**Figure 2:**
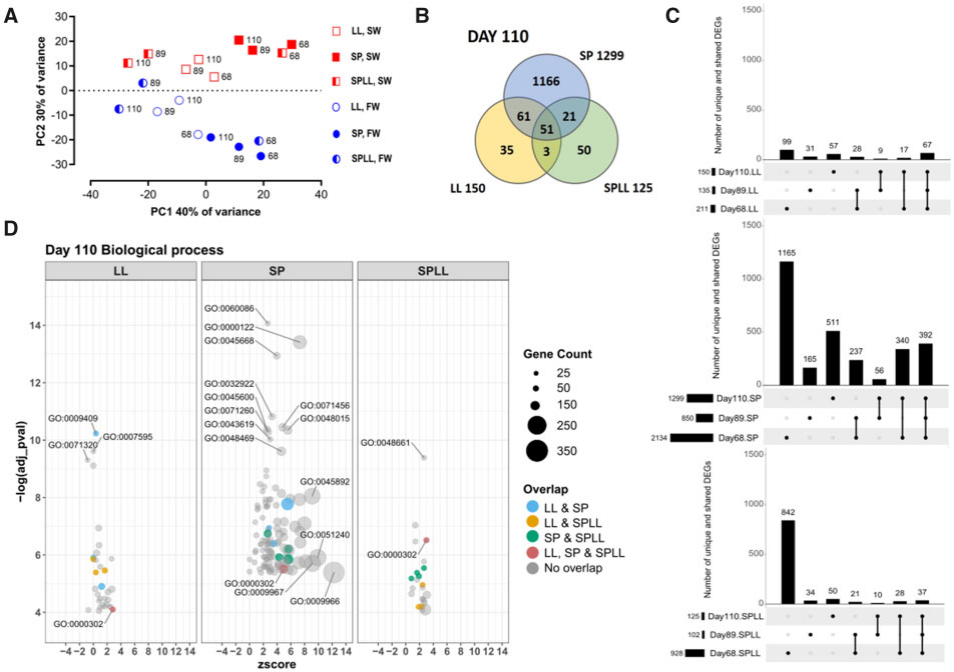
Effect of photoperiodic history on the gill transcriptomic response to SW-challenge. (A) PCA plot based on gene expression of the sampled fish. Blue indicates fish sampled from FW and red indicates fish sampled after a 24 hours SW challenge. (B) Venn diagram showing the number of genes whose expression was significantly induced or suppressed when compared the SW-challenged profile to the corresponding FW control (DEGs, *P* < 0.01, log_2_-FC>|1|) found for each treatment condition at day 110, and the degree of overlap between the treatments. (C) “Upset”-plots, indicating how the number of DEGs changed across the three latter timepoints of the experiment for each of the treatments. The bar graph shows number of unique or shared genes for the treatment group(s) indicated by the table below. (D) GO-term analysis of SW-sensitive gene expression on day 110 for the 3 photoperiod treatments; data are shown as Bubble-plots of enriched biological process (BP) GO-terms and the number of genes linked to each term. Terms enriched across groups are indicated by color. Strongly represented GO-terms are labeled. See Supplementary Figure S3 for other timepoints and GO categories, and Supplementary Tables S4–S6 for a table of GO-terms and names.

A quasi-likelihood negative binomial generalized log-linear model was used to fit the data, and nine empirical Bayes *F*-tests were run contrasting between the FW and SW sampled fish for each condition on days 68, 89, and 110 of the study. Outputs were filtered requiring a false discovery rate (FDR) of 0.01, and a log_2_-fold change of |1|. Lists of differentially expressed genes (DEGs) from each of the sampling groups were compared across time within treatments, and between treatments at the same time point. The numbers of unique and shared DEGs are summarized in the “Upset”-plots (UpSetR ver. 1.4.0) (Conway *et al.* 2017) in Figure 2.

Gene ontology analysis was performed on lists of DEGS generated by Edge R, using topGO (ver. 2.24.0) and the annotation package for the salmon genome Ssa.RefSeq.db (ver. 1.2) (https://rdrr.io/github/FabianGrammes/Ssa.RefSeq.db/), with a gill-specific gene universe. Fisher statistics and the “elim”-algorithm (Alexa *et al.* 2006) were applied, with a significance threshold of *P* < 0.05 for enrichment. Only the top 150 GO terms were included in the output. GOplot (ver. 1.0.2) (Walter *et al.* 2015) and ggplot2 (ver. 3.0.0) were used to visualized GO term enrichment. For each GO term (equation 1), positive z scores indicate over-representation of upregulated genes within the GO term, and negative *z* scores indicate downregulated genes. Before plotting, unique GO IDs were filtered for a count > 5.

(1) $$z-\mathrm{score}=\frac{\left(\#\mathrm{upregulated}\;\mathrm{genes}\;-\;\#\mathrm{downregulated}\;\mathrm{genes}\right)\;}{\sqrt{\mathrm{Total}\;\mathrm{number}\;\mathrm{of}\;\mathrm{genes}\;}}$$

### NFAT family member gene expression

From the set of expressed genes (CPM > 1 in five or more libraries), 18 genes could be identified as NFAT (5 genes), NFAT-like (12 genes), or NFAT-interacting genes (1 gene) based on their SalmoBase annotation (ICSASG_v2) (Lien *et al.* 2016; Samy *et al.* 2017). Raw count data were used to calculate mean gene expression at each sampling point for all three treatments. The gene expression of the SP treatment group was then hierarchically clustered using the R-package pheatmap (ver. 1.0.10) (row scaled by z-scores, applying Euclidian distance measures and complete linkage clustering).

### Motif analysis

Motif enrichment analysis was performed using SalMotifDB-shiny tool (https://cigene.no/tools/), described in detail elsewhere (Mulugeta *et al.* 2019). This tool accesses a database containing over 19,000 predicted transcription factor binding sites (TFBSs) found in the proximal promoter regions (−1,000/+200bp from TSS) of salmonid genes. The motif enrichment analysis utility of this tool was used to screen for the enrichment of NFAT and glucocorticoid response element (GRE) motifs in lists of DEGs.

## Results

### Phenotypic attributes

To verify that the lighting protocol produced the anticipated phenotypic response, we focused on two attributes: body condition factor (CF = 100 × wet weight/length^3^) and hypo-osmoregulation during a 24 hours SW challenge.

Over the course of the study, CF remained stable and at a similar value in both the LL and SP groups of fish (Figure 1B). Contrastingly, a pronounced decline if CF was seen over the last 3 sampling points in the SPLL fish (*P* < 0.001 for photoperiod treatment x time interaction, two way ANOVA), so that, by the end of the experiment CF was approximately 20% lower in SPLL fish than in either SP or LL fish. This decline in CF is a standard hallmark of light-induced smoltification (Björnsson *et al.* 1989; Stefansson *et al.* 2008).

At the beginning of the experiment (day 1), when all fish were acclimated to LL, exposure to a 24 hours SW challenge resulted in a plasma osmolality of 361 ± 4.7 mosmol/l. In fish that remained in LL, this response was largely stable over the course of the study, with a slight but significant decline in osmolality seen in SW-challenged LL fish at the last sampling on day 110 (343 ± 5.0 mosmol/l; *P* < 0.05 compared to day 1 by Tukey *post hoc* test).

Transfer to SP produced a sustained reduction in hypo-osmoregulatory performance in response to SW challenge (Figure 1B), with plasma osmolality at days 53 and 68 of the experiment being some 20% higher compared to day 1 (*P* < 0.0001 by Tukey *post hoc* test). Thereafter continued SP exposure was associated with an apparent recovery of hypo-osmoregulatory ability, so that by day 110 plasma osmolality values following SW challenge were not significantly higher than at day 1.

Within 28 days of return to LL following exposure to SP, hypo-osmoregulatory performance recovered to levels not significantly different from those seen at day 1 (SPLL day 68 = 344 ± 2.1 mosmol/l), and the lowest values recorded in the study as a whole were in the SPLL group at day 110 (328 ± 7.1 mosmol/l).

### RNA profile of the gill response to SW-challenge

To explore treatment effects on the overall RNA expression profile of the gills we performed a PCA analysis (Figure 2A). The first component separated samples by photoperiodic history and sampling time (40% variation explained, PC1) while the second component separated the FW from the SW-challenged fish (30% variation explained, PC2). On the PC1 axis the largest separation of data points was between early (day 68, 1 week after re-entry to LL) and late (days 89 and 110, 4, and 8 weeks after re-entering LL) sampling points for SPLL fish. This contrasted with low PC1 resolution for samples from fish in either the LL or SP control groups. The PC2 separation was most pronounced in SP control fish and less so in LL control fish. For the SP and LL groups divergence along PC2 appear independent of time. Contrastingly, in SPLL fish, PC2 resolution was dependent on time of sampling with major segregation between FW and SW samples on day 68, 1 week after re-entering LL, while at both later time points resolution between FW and SW samples was greatly reduced. Overall the PCA analysis indicates that return to LL after SP exposure dampens the transcriptional response to SW exposure.

To further investigate this effect, we compared lists of genes whose expression was significantly induced or suppressed by 24 hours SW challenge relative to the corresponding FW control fish (SW-DEGs; FDR <0.01, fold-change > ǀ1|, supplemental material S1) for the 3 photoperiod groups. At the end of the study (day 110), we found some 10-fold more SW-DEGs in SP fish than in either the LL or SPLL groups (Figure 2, B and C). Separate gene ontology enrichment tests were performed for genes responding to SW exposure on day 110 in the three photoperiod treatments (supplemental material S3 through S6). Enriched ontologies for SP fish included up-regulated transcripts associated with chromatin silencing and suppression of transcription (*e.g.*, *histone deactylase 5, transcriptional repressor p66, NFAT5*; GO : 0000122 “*negative regulation of transcription by RNA polymerase 2*”), and also with formation of stress granules, indicative of translational arrest due to cellular stress (Anderson and Kedersha 2008) (*e.g.*, *ddx6, ddx3x, roquin 1*; GO : 0010494, “*stress granule”*).

Only 51 SW-DEGs (*i.e.*, about 5% of the SP set) were shared across all three photoperiod treatments, and this shared group included genes involved in mitochondrial respiration (*e.g.*, cytochrome P450 subunits, hexokinase-1), presumably reflecting the energy demand imposed by SW challenge. Correspondingly, the only significantly over-represented BP GO-term shared across the photoperiod treatments was GO: 0000302, ‘*response to reactive oxygen species’*, encompassing six of the shared genes (Figure 2D).

While we observe a similar number of SW-DEGs on day 110 in the LL and SPLL treatments (150 and 125 genes, respectively), the overlap between these two groups was almost entirely limited to the universally responsive energy-related genes described above. LL-specific SW-DEGs on day 110 were mainly associated with metabolism and cell signaling (f. ex. GO: 0009749 “*response to glucose*,” GO: 0051591 “*response to cAMP*”). In contrast to the SP and LL groups, the SPLL group had a dramatic reduction in DEGs in response to SW between days 68 and 110 (Figure 2C). Within the group of SW-induced genes unique to SPLL on day 110, the inward rectifying K+ channels *KCNJ1* and *KCNJ5* and “junctional cadherin 5 associated” (*JCAD*, also known as *KIAA1462*) were the most strongly induced transcripts (supplemental material S2).

### Effects of SW on the expression of NFAT family members

**Figure 3:**
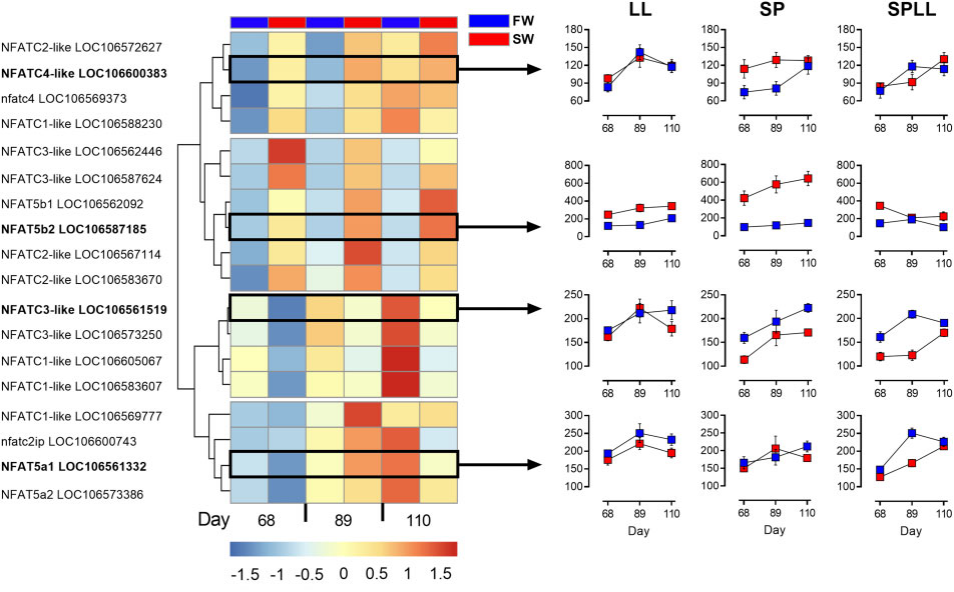
Photoperiodic history-dependent responses of NFAT family members to SW-challenge. The heatmap shows the expression of NFAT-genes (CPM) at days 68, 89, and 110 of the SP-treatment, and graphs on the right show representative profiles of selected NFAT-genes in the 3 photoperiod treatments.

The highly divergent transcriptional responses to SW, including the presence of *NFAT5* only in the list of SP-specific DEGs led us to explore further the regulation of expression among all members of the NFAT family of transcription factors (Figure 3, supplemental files S7 and S8). Clustering of response patterns across this gene family gave four distinctive patterns of regulation, represented by the four profile plots in Figure 3. The *NFAT5b* cluster (Figure 3, second cluster from the top) showed strong, SP-specific SW-induction, while weaker SP-specific SW-induction of expression was also seen in the cluster typified by *NFAT4c* (LOC106600383) (Figure 3, first cluster from the top), but only evident at earlier sampling points (days 68 and 89). Contrastingly, genes typified by *NFAT3c* (LOC106561519) showed reduced expression in SW (Figure 3, third cluster from the top). The last cluster of genes was largely SW-unresponsive across the study as a whole (Figure 3, fourth cluster from the top).

### Enrichment for NFAT- and GRE-response motifs in SW-DEGs

**Figure 4:**
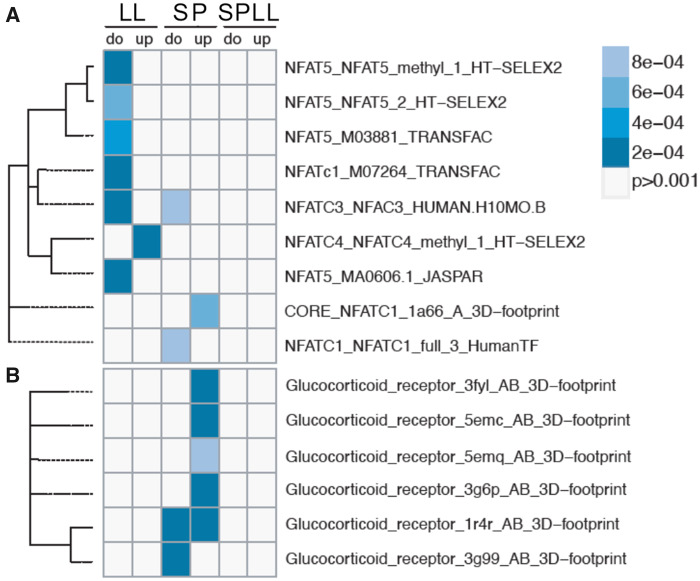
Photoperiodic history-dependent promoter motif enrichment for NFAT and glucocorticoid response elements in SW-induced transcript profiles. Panels (A and B) show the enrichment of NFAT- and GRE-transcription motifs, respectively, in up- and down-regulated genes at day 110 of the experiment (up, do, respectively), for the three different photoperiod-treatments.

We used MotifDb (Mulugeta *et al.* 2019) (https://salmobase.org/apps/SalMotifDB/) to determine how NFAT response elements are associated with SW-induced changes in gene expression (Figure 4A), focusing on changes occurring at the last sampling point (day 110) of the experiment. This revealed enrichment of seven nonredundant motifs, of which four are associated with SW-induced gene expression changes, in the LL control fish (*P ≤* 0.001). Three response elements were enriched in the SP control fish. No enrichment of NFAT elements was seen in SPLL fish at this sampling point. We also looked at presence of glucocorticoid receptor response elements (GREs, Figure 4B) due to the stress response indicated by GO-terms in the SP group, and confirmed that these were only enriched among the SW-response genes in the SP-group (Figure 4B).

## Discussion

Recently, we used RNAseq to demonstrate that photoperiodic history produces a complex suite of changes in gill function during the freshwater preparative phase of smoltification in juvenile Atlantic salmon (Iversen *et al.* 2020). Here, we have extended that analysis to consider how photoperiodic history and associated preparative changes in gill function affects the gill response to SW exposure. SP exposure dramatically impairs the ability of juvenile salmon to hypo-osmoregulate in SW and is associated with extensive changes in gill gene expression (Figure 2), including genes predicted to be regulated by the glucocorticoid pathway (Figure 4B), indicative of cellular stress. Contrastingly, exposure of LL acclimated fish to SW results in a comparatively modest osmoregulatory disturbance over 24-h, and is associated with less extensive changes in gill gene expression (Figure 2). Nevertheless, a major effect of photoperiodic history was observed in the transcriptional response of LL acclimated fish to SW, with the response profiles of fish held on LL throughout life being highly distinctive from those fish which had experienced an 8-week period of exposure to SP prior to return to LL. The diminished role of NFAT transcriptional regulation in the SW response of SPLL fish (Figure 4A) suggests that preparative effects of SP exposure reduce the involvement of pathways linked to changes in cellular tonicity or intracellular calcium levels in the response to SW.

Previous work by Lorgen *et al.* (2015, 2017) showed that in the gill the SW-induced gene *dio2a* is enriched for NFAT5 response-elements, and that expression of both *dio2a* and *NFAT5b* is SW-induced in SP-acclimated Atlantic salmon juveniles. Our RNAseq analysis confirms these findings, showing that the strongest SW-induction of *NFAT5b* is indeed seen in SP acclimated fish, as well as implicating *NFAT4* and *NFATc3* in the response. Given that this is the case, it is somewhat surprising that statistical enrichment for NFAT motifs is less pronounced within the SW-induced transcriptome of SP fish than in LL fish. We believe this may reflect a swamping of signal by large numbers of genes induced through stress-activated pathways, including but probably not limited to the corticoid axis revealed by GRE enrichment in SW-induced genes in SP fish. In support of this interpretation, the subset of SW-induced genes shared between fish in the LL and SP groups on day 110, which constitutes less than 10% of the overall SP SW-induced group (but about half of the LL SW-induced group) is highly enriched for NFAT5 elements (*P* < 0.01).

Despite the superficial similarity observed between the LL and SPLL fish in ability to hypo-osmoregulate (Figure 1B) as well as the magnitude of transcriptional responses to SW exposure (Figure 2), it is clear from the GO analysis that the SW-responses of fish in these two groups are quite distinctive. We suggest that the marked enrichment of NFAT-response elements, and in particular NFAT5, in the LL group reflects a transient activation of NFAT5-responsive genes in response to SW. By contrast, in the SPLL group there is no motif enrichment for NFAT5 nor the Ca^2+^-regulated NFATs. We interpret this lack of NFAT5 responses in SPLL as evidence for NFAT5-signaling playing a role in the activation of hypo-osmoregulation in salmon which have not developed a SW migratory phenotype. Accordingly, exposure to SP for 8 weeks prior to re-exposure to LL stimulates pre-adaptation and obviates the need for NFAT-mediated responses to SW exposure—presumably because, even in the initial phase of SW exposure, the changes in tonicity or intracellular Ca^2+^ levels in pre-adapted gill cells are comparatively modest.

The transcriptional response of the NFAT family was not limited to *NFAT5b* since we also observed SW-induction of *NFATc1* and *NFATc4* in the SP group, and photoperiodic history-dependent SW-suppression of *NFATc3* and *NFATc1* paralogous pairs in the SP and SPLL groups. In mammals, these calcium-regulated NFAT’s play important roles in immune function, but also in the development, differentiation, and function of various other cell types such as osteoclast and cardiac tissue (Hogan *et al.* 2003; Macian 2005; Ames *et al.* 2016). Changes in intracellular calcium leading to NFAT activation may conceivably arise as a result of Ca^2+^ production as a second messenger within the cell, or as a result of Ca^2+^ entry from the environment—and both these pathways are likely to be involved in osmosensing (Kültz 2012).

In addition, extracellular Ca^2+^ may affect gill function through the G-protein coupled calcium sensing receptor (CaSR), expressed in the MRCs and proposed to function as a salinity sensor in fish (Nearing *et al.* 2002; Loretz 2008; Loretz *et al.* 2009). While CaSR signal transduction has primarily been linked to cAMP-dependent signal transduction, the possibility of cross-talk with NFAT pathways is suggested by work on TNF secretion in the mammalian kidney tubule (Abdullah *et al.* 2006; Gong and Hou 2014).

Our results clearly show that NFATs are playing a minor role in SW regulated transcriptional responses in SPLL fish compared to LL and SP. This is consistent with a model where the photoperiodic treatment received (SPLL) is known to stimulate a range of smolt characteristics including improved long-term performance in SW (Saunders *et al.* 1985; Stefansson *et al.* 1991, 2008; Berge *et al.* 1995; McCormick *et al.* 1995, 2007). With the exception of day 68 (*i.e.*, the 1st week after return to LL from SP, when these fish are in a transitional state), there is no SW-induction of *NFAT5b*-expression or any other NFATs, nor is there any enrichment of NFAT-motifs in the SW-responsive transcriptome. Nevertheless, a small number of genes were uniquely stimulated by SW in the SPLL group. These included the inward rectifying potassium channel genes *KCNJ1* and *KCNJ5*, the former being ATP-regulated and the latter being G-protein regulated (Ho *et al.* 1993; Clapham 1994; Krapivinsky *et al.* 1995). Also, we find the cardiac regulatory gene junctional protein associated with coronary artery disease, known as *JCAD*. The potassium channels have been identified as key markers for SW adaptation in eels, where they have been found to be expressed in MRCs (Suzuki *et al.* 1999; Tse *et al.* 2006). JCAD is predicted to play a role in endothelial cell junctions (Akashi *et al.* 2011) and has been linked to the Hippo signaling pathway (Jones *et al.* 2018), which regulates cell proliferation and apoptosis (Halder and Johnson 2011). Both *KCNJ1* and *JCAD* show high SW-inducibility after being exposed to the photoperiod-induced smolting (S2), and they therefore represent the final activational response to SW occurring specifically in fish that have completed a FW preparative phase in response to photoperiod. Further studies to understand the impact of these genes on gill function in SW are now warranted.

## Data Availability

All relevant data and supporting information can be found within the manuscript or its supporting information, and the full transcriptomics dataset is accessible in the ArrayExpress depository, with accession number E-MTAB-8276. Supplementary material available at https://doi.org/10.25387/g3.12017100.
